# Stigmasterol accumulation causes cardiac injury and promotes mortality

**DOI:** 10.1038/s42003-018-0245-x

**Published:** 2019-01-16

**Authors:** Caroline Tao, Artem A. Shkumatov, Shawn T. Alexander, Brandon L. Ason, Mingyue Zhou

**Affiliations:** 1 Cardiometabolic Disorders Therapeutic Area, Amgen Research, South San Francisco, CA USA; 2 Comparative Biology and Safety Sciences, Amgen Research, South San Francisco, CA USA

## Abstract

Cardiovascular disease is expected to remain the leading cause of death worldwide despite the introduction of proprotein convertase subtilisin/kexin type 9 inhibitors that effectively control cholesterol. Identifying residual risk factors for cardiovascular disease remains an important step for preventing and clinically managing the disease. Here we report cardiac injury and increased mortality occurring despite a 50% reduction in plasma cholesterol in a mouse model of phytosterolemia, a disease characterized by elevated levels of dietary plant sterols in the blood. Our studies show accumulation of stigmasterol, one of phytosterol species, leads to left ventricle dysfunction, cardiac interstitial fibrosis and macrophage infiltration without atherosclerosis, and increased mortality. A pharmacological inhibitor of sterol absorption prevents cardiac fibrogenesis. We propose that the pathological mechanism linking clinical sitosterolemia to the cardiovascular outcomes primarily involves phytosterols-induced cardiac fibrosis rather than cholesterol-driven atherosclerosis. Our studies suggest stigmasterol is a potent and independent risk factor for cardiovascular disease.

## Abstract (web summary)

Tao et al. showed that stigmasterol-induced cardiac fibrosis rather than cholesterol-driven atherosclerosis determines the pathological outcomes in a preclinical model of phytosterolemia. These findings suggest that elevated concentration of phytosterols represents an independent risk factor for cardiovascular disease.

## Introduction

Cardiovascular disease remains the leading cause of death in the United States and accounts for over 15 million deaths worldwide in 2017^1^. Elevated cholesterol is the primary cause of atherosclerosis and major risk factor for cardiovascular disease. Since the 1990s, statins have been the first-line therapy for lowering low density lipoprotein-cholesterol (LDL-C) in high risk patients. In 2015, the FDA approved the first proprotein convertase subtilisin/kexin type 9 (PCSK9) inhibitor as a second line cholesterol-lowering therapy.^2–5^ Combination therapies with statins and PCSK9 inhibitors are capable of reducing LDL-C below 40 mg/dL, which is considerably lower than was previously possible^2,6,7^. Despite cholesterol levels being tightly controlled, morbidity and mortality resulting from cardiovascular disease remain substantially high. Identifying residual cardiovascular risk factors that are independent of cholesterol is an area of active research.

Understanding the pathophysiological mechanism of sitosterolemia may uncover residual risk factors of cardiovascular disease. Sitosterolemia, also known as phytosterolemia, is associated with an increased risk of cardiovascular disease. These patients respond poorly to statin therapy^8^. Phytosterolemic patients often manifest fatal myocardial infarction and sudden cardiac death at a young age^9–11^. This disorder is characterized by elevated plasma concentrations of phytosterols including β-sitosterol, campesterol, and stigmasterol^9,12^. Plasma cholesterol concentrations reported in phytosterolemic patients are highly variable, ranging from subnormal to severely elevated^12–15^. Conceivably, those reported cholesterol values may be inflated to various degrees, since standard analytical methods for measuring cholesterol are incapable of differentiating cholesterol from phytosterols species^16,17^. The increased cardiovascular disease risk observed in phytosterolemic patients is corroborated by genome-wide association studies, which link loss-of-function and gain-of-function variants of ATP-binding cassette subfamily G member 5 (*ABCG5*) and member *8* (*ABCG8)* to detrimental and beneficial cardiovascular outcomes, respectively^18–20^. *ABCG5* and *ABCG8* genes encode a heterodimer sterol efflux transporter, ABCG5/8, which plays a critical role in transporting cholesterol and phytosterols outwards across apical membranes of enterocytes and hepatocytes, thus preventing dietary phytosterols accumulation in the body^9,12^. Phytosterols share many structural similarities with cholesterol, but unlike cholesterol, phytosterols cannot be synthesized in mammalian cells. Phytosterols are solely acquired from dietary sources such as vegetable oil, soybeans, nuts, and seeds. Despite being present at comparable quantities with cholesterol in typical human diets, phytosterols are largely prevented from intestinal absorption and are effectively excreted via bile, with only trace amount of phytosterols left in healthy individual (<0.5 mg/dL plasma)^21,22^. While β-sitosterol, campesterol and stigmasterol are the three most common phytosterol species, stigmasterol concentration in healthy humans and rodents are approximately 50−100-fold lower than β-sitosterol and campesterol^23,24^. People carrying loss-of-function variants of *ABCG5* or *ABCG8* have an impaired ability to eliminate dietary sterols and this defect results in phytosterols accumulation in blood and other tissues up to hundreds fold higher than normal^12,25–28^. Interestingly, significant increases in plasma phytosterols and a greater risk for cardiovascular disease were also documented in people carrying blood *ABO* gene SNP rs657152 (*p* = 9.4 × 10^−13^)^18^.

The pathophysiological mechanisms underlying the increased risk of cardiovascular disease in phytosterolemic patients remain unclear. Current concept highlights the development of hypercholesterolemia and premature atherosclerosis in phytosterolemic patients as the cornerstones of the cardiovascular outcomes. This perception, however, is challenged by the lack of clinical benefits from statin therapies for the phytosterolemic population. Identification of principal risk factors and mechanisms that can lead to cardiovascular endpoints in phytosterolemic patients was made complicated by laboratory methodology incapable of discriminating and quantifying individual sterol species. Presence of heterogeneous genetic backgrounds with other spontaneous mutations may also contribute to cardiovascular phenotype^29–31^. It remains unclear whether cardiovascular events observed in phytosterolemic patients are a direct result of vascular atherosclerosis or primarily a cardiac focused lesion. Adding to this complexity is an established belief that consuming phytosterols-rich foods may provide cardiovascular benefits whereas emerging data warrant further investigation into the impact of phytosterols in cardiovascular health and to clarify whether phytosterols are beneficial or detrimental^32–35^. In this study, we dissect the confounding factors pertaining to phytosterolemia and elucidate a pathophysiological mechanism that links stigmasterol to cardiovascular disease.

## Results

### Stratified alterations of plasma sterols

To dissect confounding factors associated with clinical sitosterolemia and assess the impact of β-sitosterol, campesterol, stigmasterol and cholesterol to cardiovascular endpoints, we developed a mouse model of phytosterolemia using C57BL/6 mice with *Abcg5* and *Abcg8* double knockout (DKO) to compare with C57BL/6 wildtype (WT) mice^36^. Eight-week-old mice were fed a base chow diet (chow) for 12 weeks or chow supplemented with 0.2% β-sitosterol and 0.2% stigmasterol, hereafter referred to as phytosterols-rich diet (PSRD). Sterol concentrations in mouse plasma were determined by liquid chromatography with tandem mass spectrometry (LC-MS/MS), a method capable of differentiating cholesterol and individual phytosterols.

**Fig. 1:**
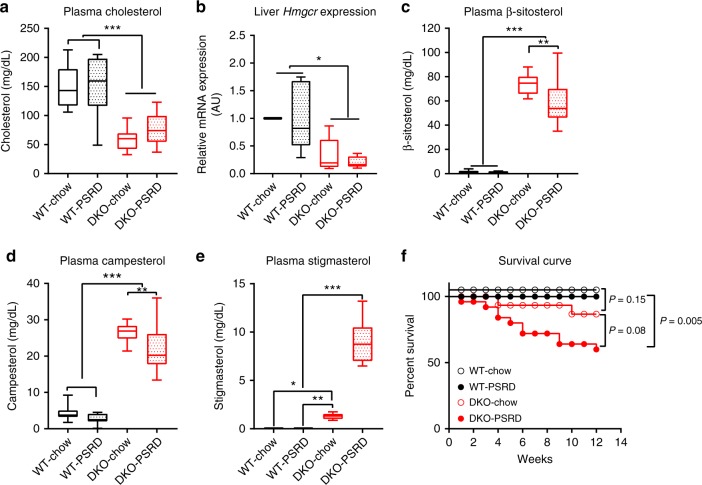
Sterol profiles in mouse model of phytosterolemia. Cohorts were designated as follows: WT-chow for wildtype C57BL/6 mice (WT) fed a base diet; WT-PSRD for WT mice fed a plant sterols rich diet (PSRD) as described in the text; DKO-chow for *Abcg5/8* double knockout (DKO) mice fed a base chow diet; and DKO-PSRD for *Abcg5/8* DKO mice fed PSRD. Plasma concentrations of cholesterol and individual phytosterols were determined by LC-MS/MS at week 12 (terminal). **a** cholesterol concentrations; **b** relative *Hmgcr* mRNA levels in liver tissues collected at week 12. β-actin was used as internal control, and gene expression was then normalized to WT-chow for comparison; **c** β-sitosterol; **d** campesterol; and **e** stigmasterol concentrations. **f** Survival curve during the 12 weeks study. No death occurred in WT-chow (*n* = 15) or WT-PSRD (*n* = 16) cohorts. Two deaths occurred in the DKO-chow (*n* = 15) cohort and 10 deaths in the DKO-PSRD (*n* = 25) cohort. Data in **a**, **c**, **d**, and **e** were analyzed using one-way ANOVA; data in **b** were analyzed using Student’s *t*-test. Data are presented using box-and-whisker plot, where the boxes encompass the first to the third quartiles, inside the box the horizontal line shows the median, and the whiskers are the maximum and minimum observation. *p < 0.05, **p < 0.005, ***p < 0.0001

Figure 1 shows individual sterol concentrations in plasma samples at week 12. Cholesterol concentrations between the two WT cohorts were comparable. In contrast, DKO-chow and DKO-PSRD mice showed 60% (*p* < 0.0001) and 49.4% (*p* < 0.0001) reduction in cholesterol, respectively, relative to WT-chow (Fig. 1a). No differences were observed between the WT-PSRD and DKO-PSRD cohorts when a clinical chemistry analyzer (COBAS INTEGRA 400 plus) was used to measure plasma cholesterol. However, LC-MS/MS analyses of the same mice plasma uncovered a 64% cholesterol reduction (*p* < 0.0001) in the DKO-PSRD cohort in comparison to WT-PSRD group (Supplementary Fig. 1). Comparing these two cholesterol quantification methods highlights a major limitation of clinical chemical analyzers as a tool for measuring cholesterol in phytosterolemic patient samples. Reductions of plasma cholesterol in DKO cohorts fed chow and PSRD were corroborated by reductions of hepatic mRNA expression of HMG-CoA reductase (*Hmgcr)*, the rate-limiting enzyme for de novo cholesterol synthesis (Fig. 1b). Plasma levels of β-sitosterol, campesterol, and stigmasterol were quantified (Fig. 1c−e). The three phytosterol species remained very low in the two WT cohorts, thus indicating ABCG5/8 transporter functionality. All three phytosterol species were significantly (*p* < 0.0001) higher in the two DKO cohorts, indicating development of phytosterolemia. The overall plasma sterol profiles differed between the two DKO cohorts. Specifically, DKO-chow mice had the highest plasma β-sitosterol (73.86 ± 2.28 mg/dL) and campesterol (26.42 ± 0.68 mg/dL) concentrations, but a much lower stigmasterol level (1.28 ± 0.08 mg/dL) than the DKO-PSRD cohort. In contrast, DKO-PSRD cohort β-sitosterol (59 ± 4.56 mg/dL) and campesterol (21.81 ± 1.50 mg/dL) concentrations were 20% (*p* < 0.005) and 17.5% (*p* < 0.05) lower respectively, but its stigmasterol concentration (8.92 ± 0.50 mg/dL) was 6.9-fold higher (*p* < 0.0001) relative to the DKO-chow cohort. Of note, while DKO-PSRD mice had a net stigmasterol increase of 7.69 mg/dL over DKO-chow cohort, this increase in stigmasterol did not account for the mass reduction of β-sitosterol and campesterol in combination (19.47 mg/dL net reduction) in the DKO-PSRD cohort. These distinct sterol profiles among the four cohorts developed as early as three weeks on chow or PSRD (Fig. 5, Supplementary Fig. 1). Since the DKO-PSRD cohort featured the largest fold increase in circulating stigmasterol, and DKO-chow mice had the highest β-sitosterol and campesterol accumulation, we hereafter refer to the DKO-PSRD cohort as stigmasterolemic and DKO-chow cohort as sitosterolemic.

### High mortality and cardiac dysfunction

The stigmasterolemic cohort (DKO-PSRD) experienced 40% mortality by week 12, which was higher than the WT cohorts and more aggravated than the DKO-chow mice (Fig. 1f). Results from a pilot study led us to anticipate increased mortality in the stigmasterolemic cohort. For this reason, the number of mice enrolled into the DKO-PSRD cohort was increased to ensure the study was correctly powered (Supplementary Table 1). The sitosterolemic cohort (DKO-chow) had 13% mortality at week 12, and no deaths occurred in either of the two WT cohorts. There were no significant differences in food consumption, body weights, or heart/brain weight ratios between the stigmasterolemic and the sitosterolemic cohorts verses the WT cohorts.

**Fig. 2:**
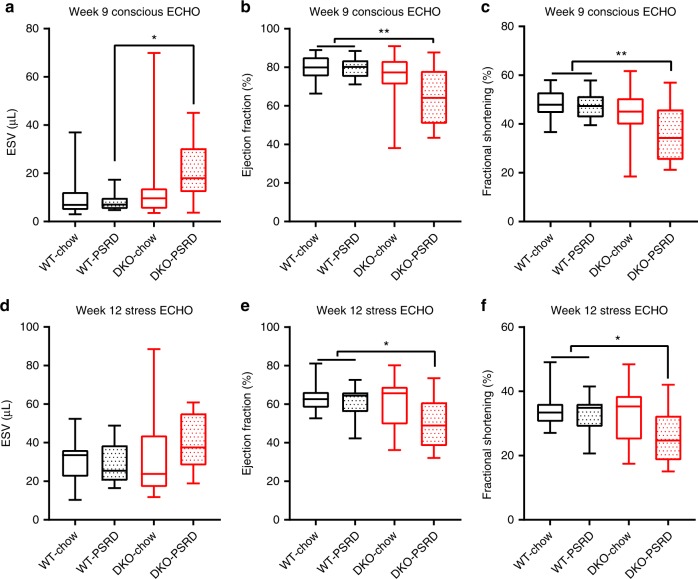
Cardiac function measurements using echocardiography (ECHO). ECHOs were performed at weeks 9 and 12 with normal conscious condition (**a−c**) and dobutamine-induced stress conditions (**d−f**). **a**, **d** End systolic volume (ESV); **b**, **e** LV ejection fraction; **c**, **f** fractional shortening. WT-chow (*n* = 15), DKO-chow (*n* = 13), WT-PSRD (*n* = 16), DKO-PSRD (*n* = 16). Data were analyzed using one-way ANOVA and presented using box-and-whisker plot, where the boxes encompass the first to the third quartiles, inside the box the horizontal line shows the median, and the whiskers are the maximum and minimum observation. *p < 0.05, **p < 0.005, ***p < 0.0001

Left ventricular (LV) function was determined at week 9 and week 12 (terminal) using echocardiography (ECHO)^37,38^. Week 9 ECHO was performed on unanesthetized (conscious) mice. Week 12 ECHO was performed on anesthetized mice administered dobutamine—a β1 adrenergic agonist—as a cardiac stress inducer. Dobutamine is used as an alternative to treadmill exercise to assess cardiac function at peak performance (stress ECHO)^37,39^. Both the stigmasterolemic (DKO-PSRD) and the sitosterolemic (DKO-chow) cohorts exhibited increased end systolic volumes (ESV) relative to WT-PSRD at week 9 (Fig. 2). These observations were supported by concurrent increases in the systole left ventricular internal diameter (LVID’s) (Supplementary Fig. 2). The stigmasterolemic cohort showed a 62% increase in ESV over WT-PSRD (*p* < 0.05) and a 21.6% increase verses DKO-chow at week 9 (Fig. 2a). Consistent with increases in ESV in the two phytosterolemic cohorts were decreases in LV ejection fraction and fractional shortening relative to WT cohorts (Fig. 2b, c). There are no significant differences in interventricular septal (IVS’d and IVS’s), end diastolic volume (EDV), heart rate, and LVID’d at week 9 when measured by conscious ECHO (Supplementary Fig. 2). The deterioration of cardiac functions observed during both conscious and stressed states occurred predominately and consistently in stigmasterolemic mice (DKO-PSRD). These functional impairments in stigmasterolemic mice collectively indicate a reduction in the pumping efficiency of the heart and correlate with the high mortality observed in this cohort (Fig. 1f).

### Cardiac fibrosis and inflammation without atherosclerosis

**Fig. 3:**
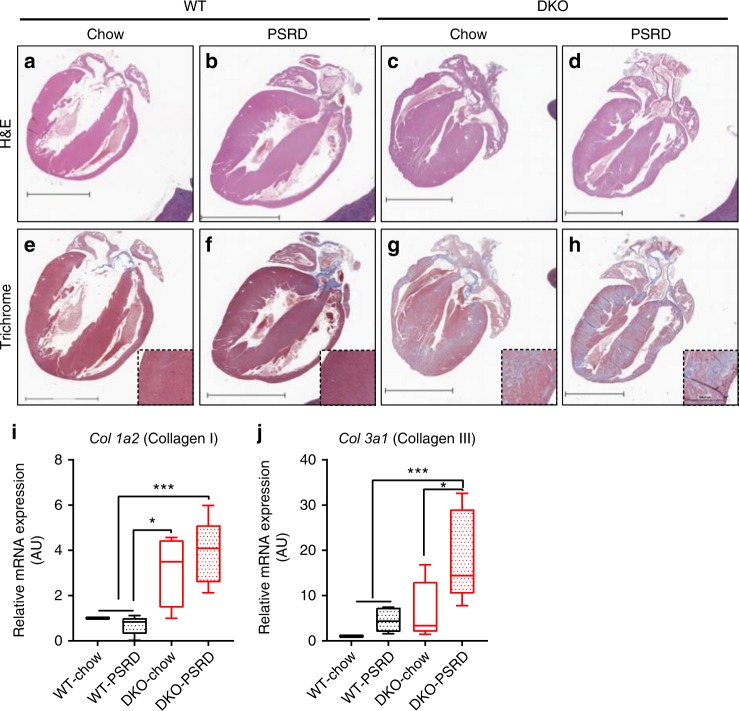
Evidence of cardiac fibrosis in survived mice at week 12. Heart tissue sections underwent H&E staining (**a−d**) and trichrome staining (**e−h**) with 10X magnification of LV shown in the lower right inset image. A single representative section for each cohort is shown. Scale bar equal 4 mm (**i−j**) qPCR was used to measure relative mRNA expression of fibrosis markers, *Col 1a2* and *Col 3a1*, in heart tissues excised at week 12. β−actin was used as an internal control, and gene expression was normalized to WT-chow for comparisons. Data in **i**, **j** were analyzed using Student’s *t*-test, *n* = 5 for each group. Data are presented using box-and-whisker plot, where the boxes encompass the first to the third quartiles, inside the box the horizontal line shows the median, and the whiskers are the maximum and minimum observation. *p < 0.05, **p < 0.005, ***p < 0.0001

Histopathology examination of isolated heart tissues revealed severe cardiac fibrosis in stigmasterolemic (DKO-PSRD) and sitosterolemic (DKO-chow) mice at week 12. WT mice, regardless of diet, showed no evidence of cardiac fibrosis (Fig. 3a−h). Trichrome staining revealed greater collagen deposition in stigmasterolemic mice than in sitosterolemic mice (Fig. 3e−f). Myocardial fibrosis was distributed diffusely throughout the left and right ventricles and interventricular septum without predilection for any particular region in the hearts of stigmasterolemic and sitosterolemic mice. This distribution pattern of collagen accumulation without particular predilection for subendocardial myocardium suggests a non-ischemic cause of fibrosis^40^. Collagen type I alpha chain (*Col 1a2*) and collagen type III alpha1 chain (*Col 3a1*) mRNAs were quantified in collected heart tissues^41^. Quantitative-PCR analyses showed a significant (*p* < 0.05) increase of *Col 1a2* and *Col 3a1* expression, with the highest mRNA level detected in the stigmasterolemic cohort (Fig. 3i–j). Stigmasterolemic mice had 2.3-fold higher *Col 3a1* expression than the sitosterolemic mice (Fig. 3j). Increased collagen content and pronounced cardiac fibrosis in heart tissues from DKO mice were evident within three weeks of following PSRD feeding (Fig. 5e–f). These results substantiate histopathological image assessments. Histopathologic evaluations of livers and spleens did not detect fibrosis (Supplementary Fig. 3).

**Fig. 4:**
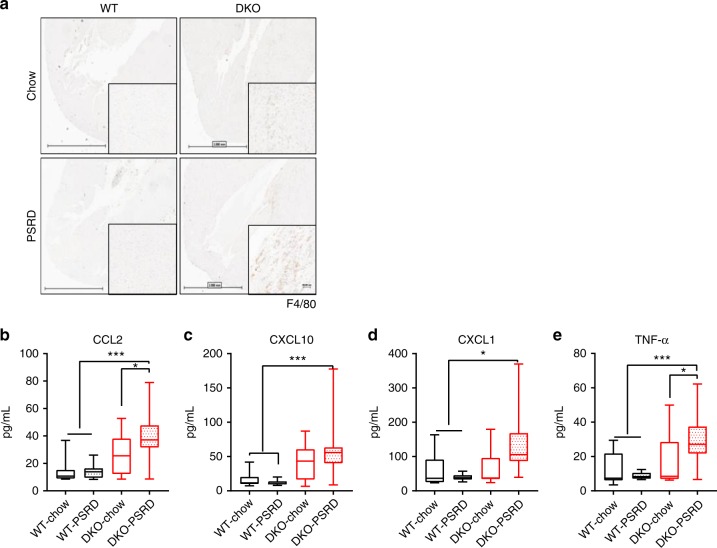
Macrophage infiltration in the heart tissues and plasma levels of proinflammatory cytokines. **a** Macrophage infiltration into mouse heart tissues at week 12 visualized by F4/80 staining with 20× magnification of the interventricular septum region shown in the lower right corner. Scale bar equals 2 mm and 60 µm. A single representative section for each cohort is shown. **b**–**e** Plasma cytokines were determined by electrochemiluminescence assay at week 3: **b** CCL2; **c** CXCL10; **d** CXCL1; and **e** TNF-α. WT-chow (*n* = 10), DKO-chow (*n* = 12), WT-PSRD (*n* = 9), DKO-PSRD (*n* = 22). Data were analyzed using one-way ANOVA and presented using box-and-whisker plot, where the boxes encompass the first to the third quartiles, inside the box the horizontal line shows the median, and the whiskers are the maximum and minimum observation. *p < 0.05, **p < 0.005, ***p < 0.0001

Examination of isolated heart tissues uncovered pronounced infiltration of F4/80 positive macrophages in stigmasterolemic mice (DKO-PSRD) and relatively lesser presentation in sitosterolemic mice (DKO-chow) (Fig. 4a). Macrophages were present in higher numbers in DKO-PSRD mice hearts. These macrophages overlapped with areas of fibrosis and degenerating cardiomyocytes throughout the heart muscle, particularly in the ventricles and interventricular septum from apex to the base of the heart. No noticeable macrophage infiltration was observed in WT mice on either diet. We reasoned accumulation of phytosterols, particularly stigmasterol, may have triggered an acute inflammatory response in the early phase of the study^42,43^. Indeed, measuring plasma cytokines at week 3 revealed a consistent increase in proinflammatory cytokines CCL2, CXCL10, CXCL1, and TNFα in sitosterolemic mice and to a much greater extent in stigmasterolemic mice (Fig. 4b−e).

Despite pronounced cardiac fibrosis and macrophage infiltration in phytosterolemic mice, there was no evident atherosclerotic plaque formation, lipid-loaded macrophages or foam cells in the aortic root and descending aorta of sitosterolemic and stigmasterolemic mice (Supplementary Fig. 4). The absence of atherosclerosis was consistent with the hypocholesterolemic state of the phytosterolemic mice. These findings indicate that the observed cardiac lesions and high mortality rate in the stigmasterolemic cohort were not caused by cholesterol-driven atherosclerosis.

### Prevention of phytosterols-induced cardiac fibrogenesis

**Fig. 5:**
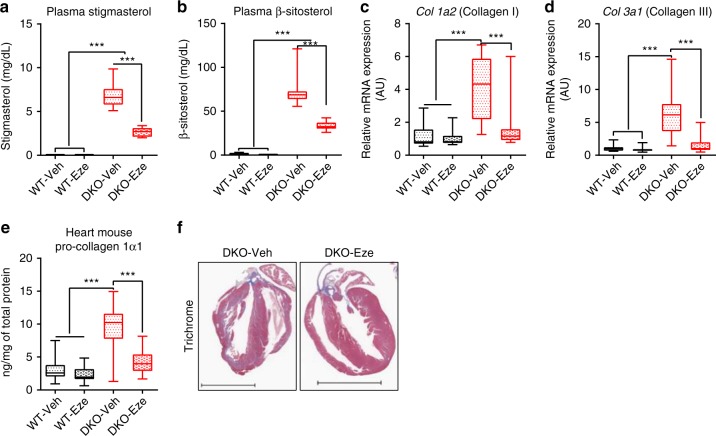
Ezetimibe restricted phytosterolemia development and prevented fibrogenesis in *Abcg5/8* knockout mice fed PSRD. Wild type C57BL/6 mice (WT; *n* = 30) and *Abcg5/8* DKO mice (DKO; *n* = 30) were fed PSRD. A subset of 15 mice from each genotype were administered 10 mg/kg ezetimibe (Eze) daily via p.o. beginning at day 0. The remaining 15 mice of each genotype received vehicle. **a** Plasma stigmasterol and **b** β-sitosterol concentrations were determined by LC-MS/MS at week 3. **c**, **d** qPCR was used to measure relative mRNA expression of fibrosis markers, *Col 1a2* and *Col 3a1*, β-actin was used as an internal control for qPCR, and gene expression were normalized to WT vehicle (Veh) mice for comparisons. **e** Pro-collagen 1 contents determined by ELISA in heart tissues excised at week 3. WT-Veh (*n* = 15), DKO-Veh (*n* = 15), WT-Eze (*n* = 15), DKO-Eze (*n* = 13). **f** Representative trichrome staining of heart tissues of DKO-Veh and DKO-Eze treated group excised from a separate three-week PSRD study, scale bar equal 4 mm. Data in **a,** **b**, **e** were analyzed using one-way ANOVA and **c**, **d** were analyzed using Student’s *t*-test. Data are presented using box-and-whisker plot, where the boxes encompass the first to the third quartiles, inside the box the horizontal line shows the median, and the whiskers are the maximum and minimum observation. *p < 0.05, **p < 0.005, ***p < 0.0001

*ABCG5* and *ABCG8* are predominately expressed in the intestine but not in the heart or vasculature across species, thus it is improbable that cardiac injuries observed in *Abcg*5/*8* double knockout mice were caused simply by the mere absence of ABCG5/8 transporter in the heart. We hypothesize that cardiac dysfunctions in stigmasterolemic mice are a direct result of phytosterols accumulation in the blood and heart tissues. To separate the impact of phytosterols accumulation from the lack of functional ABCG5/8 transporters itself, a separate three-week study was performed. DKO and WT mice were fed PSRD and concomitantly, half of the mice of each genotype were administered ezetimibe or vehicle at 10 mg/kg per day via oral gavage. Ezetimibe antagonizes Niemann-Pick C1-Like protein 1 (NPC1L1), a sterol influx transporter expressed in mouse intestine^44,45^. Administrating ezetimibe for three weeks significantly restricted the development of phytosterolemia in DKO mice (DKO-Eze), with 61% stigmasterol reduction (*p* < 0.0001) and 54.6% β-sitosterol reduction (*p* < 0.0001) versus DKO mice administered vehicle (Fig.  5a−b). Increases in cardiac *Col 1a2* and *Col 3a1* gene expression (Fig. 5c−d), pro-collagen 1 protein expression (Fig. 5e), and pronounced cardiac fibrosis visualized by trichrome staining (Fig. 5f) in vehicle-treated phytosterolemic mice were evident in heart tissues following three weeks on PSRD. Ezetimibe administration effectively prevented these changes. These results indicate phytosterols accumulation and not the lack of ABCG5/8 transporter itself is responsible for the observed cardiac fibrotic phenotype.

### Sterol effects on cell viability

**Fig. 6:**
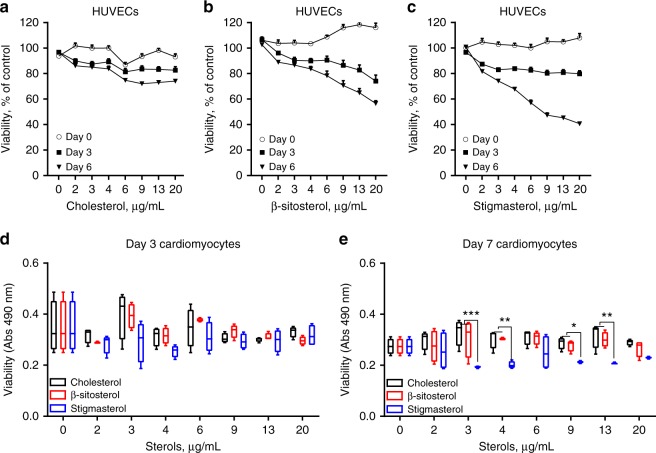
Phytosterols reduced in vitro viability of HUVECs and iPSCs-derived cardiomyocytes. HUVECs were cultured in normal culture medium supplemented with increasing concentrations of **a** cholesterol, **b** β-sitosterol, or **c** stigmasterol as indicated. Cell viability was measured at indicated time points using CellTiter 96 Aqueous One Solution. Cell viability of iPSCs-derived cardiomyocytes were measured **d** 3 days and **e** 7 days after cultured in medium enriched with various concentration of sterols. Each data point represents means ± SEM of three independent replicates (**a**–**c**) and box-and-whisker plot, where the boxes encompass the first to the third quartiles, inside the box the horizontal line shows the median, and the whiskers are the maximum and minimum observation (**d**, **e**). Data in **d**, **e** were analyzed using two-way ANOVA. *p < 0.05, **p < 0.005, ***p < 0.0001

The detrimental cardiac phenotype observed in stigmasterolemic mice (DKO-PSRD) suggests stigmasterol is a more potent cardiac toxic molecule than β-sitosterol and campesterol. To test this inference, in vitro experiments were performed to evaluate the impact of each individual sterol species on the viability of cultured human umbilical vein endothelial cells (HUVECs) and iPSCs-derived cardiomyocytes (Fig. 6). Viability of HUVECs cultured in the presence of supplemented cholesterol, β-sitosterol, or stigmasterol were measured repeatedly (Fig. 6a−c). Viability was greatly compromised by stigmasterol, and to a lesser degree, by β-sitosterol. Cholesterol modestly affected cell viability to a lesser extent than the two phytosterols evaluated. Viability of iPSCs-derived cardiomyocytes was also reduced by stigmasterol, but not by cholesterol or β-sitosterol (Fig. 6d, e). These in vitro viability results support a stronger cytotoxicity of stigmasterol over β-sitosterol and cholesterol to cardiovascular relevant cell lines.

## Discussion

By dissecting confounding factors that conceal the pathophysiological mechanism linking clinical sitosterolemia to cardiovascular outcomes, we demonstrated that stigmasterol accumulation can lead to cardiac injury and promote mortality in a rodent model of phytosterolemia. Stigmasterol-induced cardiac detriments occurred in the context of considerably reduced cholesterol levels and primarily involved cardiac fibrosis but not atherosclerosis. These findings challenge the current perception of sitosterolemia-associated cardiovascular events—events believed to be driven by hypercholesterolemia and premature atherosclerosis. The body of evidence produced by our studies suggest that stigmasterol is an extremely potent and independent risk factor for cardiovascular disease, and that the pathological mechanisms linking sitosterolemia and cardiovascular disease primarily involve cardiac fibrosis and inflammation rather than cholesterol-driven atherosclerosis.

It is known that clinical manifestations of phytosterolemia are notably different from hypercholesterolemia, and statin therapies are ineffective in phytosterolemic patients. Despite these observations, hypercholesterolemia and premature atherosclerosis are still considered the primary pathological mechanisms leading to cardiovascular outcomes in phytosterolemic patients^46,47^. It was recently reported that several patients initially diagnosed and treated for hypercholesterolemia were later found to be phytosterolemia^48,49^. Prior publications with *Abcg5/8*-deficient mice reported phytosterols-related systemic toxicity and cardiomyopathy, but neither identified the principal causative sterol species nor clarified the role of cholesterol in the observed phenotype^29,31^. There are no current reports, which excluded cholesterol as a contributing factor to sitosterolemia-associated cardiovascular events.

We optimized a preclinical phytosterolemia model to investigate the role of individual sterol species. Using a combination of genetic modeling, dietary modulation, mass spectrometry determination of individual sterol, and pharmacological intervention led to several important new insights. In summary, phenotyping four distinct mouse cohorts reveals a decrease in LV cardiac function (Fig. 2); severe cardiac fibrosis (Figs. 3, 5); pronounced macrophage infiltration in the heart tissue; systemic inflammatory response (Fig. 4); and accelerated mortality rate (Fig. 1f), all these findings occurred in stigmasterolemic (DKO-PSRD) mice in the context of a near 50% reduction in plasma cholesterol (Fig. 1). Evidence that could point to the presence of atherosclerosis was not observed in histopathological samples collected from phytosterolemic mice (Supplementary Fig. 4) and this is consistent with their hypocholesterolemic state. Reducing intestinal sterol absorption with ezetimibe prevented cardiac fibrogenesis despite the lack of ABCG5/8 transporter functionality (Fig. 5). Overall, the phenotype observed in stigmasterolemic mice is distinct from that of hypercholesterolemic mice developed by feeding *Ldlr* and/or *ApoE* knockout mice high cholesterol diets^50–52^. The cardiac lesions documented in stigmasterolemic mice appear more similar to chronic heart failure rather than vascular atherosclerosis^53^. Cardiac injury and severe mortality mostly correlate with stigmasterol accumulation, and to a lesser extent, with β-sitosterol and campesterol; no correlation with cholesterol.

To our knowledge, this is the first study demonstrating stigmasterol as an independent and potent causative factor of cardiac fibrosis and dysfunction with increased mortality. Circulating stigmasterol is not routinely measured in clinics. Perhaps this is in part due to stigmasterol low concentration and limited accessibility to instruments capable of measuring stigmasterol levels in blood^24,54^. The mechanism whereby stigmasterol causes cardiac fibrosis is not fully understood. Basal stigmasterol is considerably lower than β-sitosterol and campesterol in healthy humans, but could increase up to 2 mg/dL level during disease conditions^28^. Stigmasterol structure differs from that of β-sitosterol and campesterol by the presence of Δ-22 double bond that confers increased hydrophobicity and decreased membrane fluidity^55^. An extreme hydrophobicity feature of stigmasterol, in addition to its low propensity to be esterified, may contribute to its potential to be proinflammatory and to adversely alter cellular membrane dynamics, thus having a stronger cytotoxic impact than β-sitosterol and campesterol. Perhaps the cytotoxic properties of stigmasterol created evolutionary pressures that ultimately shaped robust protective mechanisms to guard against stigmasterol accumulation in humans. The stigmasterol concentration in the stigmasterolemic mice used in our study was 8.9 mg/dL, which is near the general range of sitosterolemic patients^12,28^. This documented stigmasterol elevation is merely on par with basal levels of β-sitosterol and campesterol in wildtype mice and healthy humans, and orders of magnitude lower than cholesterol. Despite this, stigmasterol accumulation of approximately 9 mg/dL in the blood was sufficient to cause severe cardiac fibrosis and increase mortality within a few weeks, even though the mice were presumably benefiting from a considerable reduction in cholesterol. Based on the results of this study and studies done in other labs^29,30,42,56^, we propose three plausible mechanisms to explain observed cardiac fibrosis in stigmasterolemic mice: (1) phytosterols accumulated in blood and heart tissue adversely perturb cardiomyocyte membrane dynamics and induce cytotoxicity; (2) enhanced macrophage infiltration to the heart along with a pronounced inflammatory response promote fibrogenesis; and (3) phytosterols-induced macrophage death in heart tissue via necroptosis and apoptosis exacerbates inflammation and fibrosis. The extensive cardiac fibrosis uncovered in the stigmasterolemic DKO-PSRD cohort leads us to think the aggressive mortality phenotype of this cohort may have been caused by the development of malignant ventricular arrhythmia that resulted from cardiac fibrosis. In this view, stigmasterol represents a potent residual risk factor for heart disease by inducing cardiac fibrosis and not by an atherogenic mechanism. Studies are underway to validate epidemiological and clinical association of phytosterols with cardiovascular disease risk, and to understand the prevalence of elevated phytosterols in cardiovascular disease patients.

This study underscores the need to increase awareness of the potential detriments of consuming phytosterols-rich diets, since current clinical strategy for a healthy heart recommends consuming diets high in phytosterols to lower cholesterol^57,58^. Discoveries presented herein raise important safety concerns for people with undiagnosed defects in their sterol absorption pathway. This study also highlights the importance of early diagnosis of phytosterolemia and need to differentiate this disorder from hypercholesterolemia, as phytosterolemic patients are exposed to a higher risk of CV events. Current treatments available to phytosterolemic patients include cholesterol-lowering therapeutics, statins, and ezetimibe. The bile acids sequestrant, cholestyramine, is also recommended. In addition, clinical guidelines advise phytosterolemic patients to reduce cholesterol and phytosterols in their diets. These and other available therapies are not able to adequately meet the medical need to ameliorate phytosterolemia. Clinical studies show statins have little effect on sitosterolemic patients and may even increase plasma level of phytosterols^59^. While NPC1L1 transporter plays a significant role in the absorption of phytosterols, evidence from clinical and preclinical studies (Fig. 5) consistently shows that elevated plasma levels of phytosterols resulting from loss-of-function mutations of the ABCG5/8 transporter cannot be fully restored to the normal baseline merely by inactivating NPC1L1. It was reported that ezetimibe treatment in sitosterolemic patients only partially reduced plasma concentrations of β-sitosterol (by 21−51%), campesterol (by 24−50%) and stigmasterol (by 27−41%)^60,61^. Dietary restriction may provide approximately 30% reduction in plasma phytosterols for sitosterolemic patients, and is difficult to accomplish because phytosterols are found in almost every plant-based food^34^. We propose that new therapies that are more effective in lowering phytosterols, particularly in depleting stigmasterol from the body, represent a valuable therapeutic strategy for reducing residual risk of cardiovascular disease in the post-PCSK9 inhibitor era of tightly controlled cholesterol.

## Methods

### Animals

All research protocols were reviewed and approved by the Amgen Institutional Animal Care and Use Committee. All mice in these studies were group housed in an AAALAC, International accredited facility, and cared for in accordance with the Guide for the Care and Use of Laboratory Animals, 8th Edition. Eight week old male CB57B/6-*Abcg*5 and *Abcg*8 double knock out (B6;129S6-Del(17Abc5-Abc8)1Hobb/J) mice (*Abcg*5/*8* DKO) were purchased from Charles River^36^. Housed in individual ventilated cage system on an irradiated corncob bedding (Envigo Teklad 7097). Lighting in animal holding rooms was maintained on 12:12 h light:dark cycle, and the ambient temperature and humidity range was at 68 to 79 °F and 30–70%, respectively. Animals had ad libitum access to irradiated pelleted diet (Envigo 2020X Teklad global soy protein-free extruded rodent diets) or 0.2% β-sitosterol/0.2% stigmasterol-enriched diet (PSRD) (Envigo Teklad TD 140478), and reverse-osmosis chlorinated (0.3–0.5 ppm) water via an automatic watering system. Cages were changed biweekly inside an engineered cage changing station. For ezetimibe administration, eight-week-old male *Abcg5/8* DKO mice and WT age-matched controls all had ad libitum access to PSRD when housed. Vehicle or 10 mg/kg ezetimibe (Fisher, 50-753-2773, Cat#163222-33-1) were administered daily via oral gavage for three weeks. Tissue and plasma samples were collected at week 3.

### Cell lines

Human umbilical vein endothelial cells (HUVECs) were purchased from ATCC (ATCC CRL-1730), cultured in F12K (ATCC No.30-2004) supplemented with 0.1 mg/mL heparin (sigma H4784-250MG), 0.03 mg/mL endothelial cell growth supplement (ECGS) (Sigma E2759), 10% FBS (Gibico, 16000044), and 1% penicillin/streptomycin (Gibico 10378016), at 37 °C in 5% CO_2._

iPSCs-derived cardiomyocytes (iCell Cardiomyocytes^2^) were purchased from Cellular Dynamics international (R1017), plated and cultured following manufacture’s recommendation in the provided user’s guide. Briefly, cells were seeded in gelatin coated 96 well plate in seeding medium at seeding density of 1.56 × 10^5^ cells/cm^2^, 4 h post-seeding, cells were switched to maintenance medium at 37 °C in 5% CO_2_. Medium were replaced every other day until synchronized beating of cardiomyocytes can be observed.

### Echocardiogram

Non-invasive echocardiograms were obtained from conscious mice using a VisualSonics Vevo 2100 imaging system, equipped with an imaging transducer. A commercially available depilatory cream (e.g., Nair) was applied for 1 min after shaving and wiped away to remove hair in the thorax area. The thoraxes of mice were wiped with water, and then mice were manually restrained for imaging. Sonography gel was applied to thoraxes, and two-dimensional targeted M-mode imaging was obtained from the short-axis view at the level of the papillary muscle.

To obtain echocardiograms under induced stress conditions, mice were anesthetized using isoflurane (1−5% to maintain heart rate of 450 BMP) and taped on ultrasound stage. Thorax area was shaved and wiped clean with Nair and water. Up to 2 mg/kg body weight dobutamine (Novation. LLC, NDC 0409-2344-02,) was administered via IP injection to increase cardiac pacing. Two-dimensional targeted M-mode imaging was obtained from the short-axis view at the level of the papillary muscle before and after injection.

### LC-MS/MS

Mouse plasma samples were analyzed using liquid chromatography with tandem mass spectrometry (LC-MS/MS, performed by Metabolon Inc., Durham, NC) for the contents of the following sterols (after hydrolysis of sterol esters): cholesterol, β-sitosterol, campesterol, and stigmasterol. Samples analyses were carried out in a 96-well plate format containing two calibration curves and six QC samples (per plate) to monitor method performance. The mass spectrometer was operated in selected ion monitoring (SIM) mode. The masses of the extracted ions were 458.4 (cholesterol), 382.4 (campesterol), 396.4 (β-sitosterol), and 523.2 (stigmasterol).

### Immunohistochemistry

Mice were anesthetized using isoflurane followed by cervical dislocation (*n* = 5 per group). Tissues were excised and fixed in 1× PBS buffered with 10% formalin overnight and then transferred to 70% ethanol. Following paraffin embedding, tissue sections were stained with hematoxylin and eosin (H&E) (Biocare IPCS5006G20), and trichrome using standard protocols. A subset of sections were deparaffinized, rehydrated, treated with peroxidase block and protein block. Sections were also stained with rat anti-F4/80 antibody (1:2000, BioRad MCA497R/Lot 1014) for 60 min, washed, followed by incubation with secondary antibody (Rat 1-Step Polymer, Biocare BRR4016L). Additional sections were treated with chromogen 3′-diaminobenzidine (DAB, DAKO 3468) and counterstained with hematoxylin. Images were obtained using Aperio Digital Pathology Slide Scanner (Leica Biosystems) and Aperio ImageScope v12 software.

### Real-time quantitative PCR

Total RNA was isolated from mouse tissues using RNeasy Mini Kit (Qiagen, Venlo, Netherlands). First-strand cDNA was synthesized using High Capacity cDNA Reverse Transcription Kit (Applied Biosystems, Foster City, CA) according to the manufacturer’s instruction from 2 µg of RNA. Real-time quantitative PCR of *Hmgcr* (Mm01282499_m1), *Col 1a2* (Mm00483888_m1), *Col 3a1* (Mm01254476_m1) was done with the TaqMan real-time PCR system (Applied Biosystems, Foster City, CA), using *β-actin* (Mm00607939_s1) as an internal control for normalization. PCR reactions were performed on a QuantStudio 7 Flex.

### Plasma cytokine

Inflammatory cytokines were measured at week 3 using the commercially available multiplex panels V-PLEX Plus Mouse Cytokine 19-plex kit (MSD K 15255G) following manufacturer’s instruction.

### Mouse pro-collagen 1α1 ELISA

Measurement of pro-collagen 1α1 in heart tissue was performed using the Mouse Pro-Collagen I alpha 1 CatchPoint SimpleStep ELISA Kit from Abcam (ab229425). Tissues were homogenized, after protein quantification (Pierce BCA protein assay kit, Thermo Scientific 23225) 100 µg of total protein from each sample were assayed according to manufacturer’s instruction. Fluorescence were measured at 530/590 using a 96-well plate reader (Tecan Infinite M1000).

### In vitro cell proliferation assay

Cell proliferation assay was performed according to manufacturer’s instruction using CellTiter 96 Aqueous One Solution method (Promega G3580). Cell were incubated with Aqueous One Solution containing MTS and an electron coupling reagent PES for 60 min at 37 °C with 5% CO_2_. Absorbance were measured at 490 nm using a 96-well plate reader (Tecan Infinite M1000).

### Quantification and statistical analysis

All data are reported as the mean ± SEM. The differences between the mean values were tested for statistical significance by one-way ANOVA or two-tailed Student’s *t-*test as indicated in figure legends.

## Supplementary information

Supplementary Information

## Data Availability

All data generated and analyzed during this study are included in this article and supplementary information files. Additional data and information about this study are available from the corresponding author on request.
