# Supplementary material for: Stigmasterol accumulation causes cardiac injury and promotes mortality
Source: Commun Biol. 2019 Jan 16;2:20. doi: 10.1038/s42003-018-0245-x (PMC6335236; doi:10.1038/s42003-018-0245-x)
Supplement: Supplementary file 1 — Supplementary Information [file 42003_2018_245_MOESM1_ESM.pdf]

SUPPLEMENTARY INFORMATION

Supplementary Figure 1

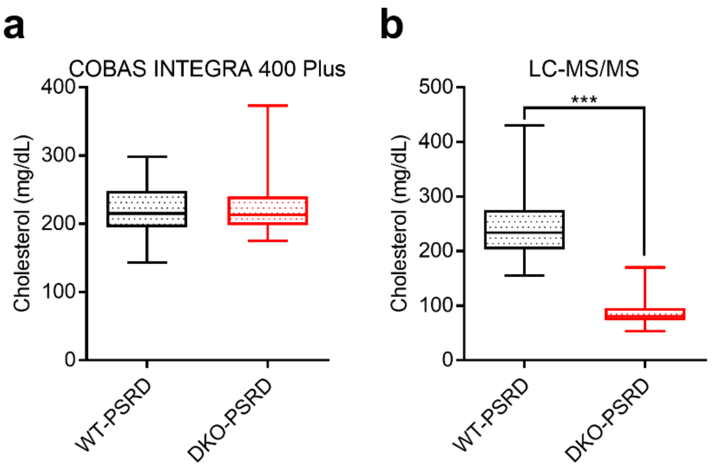

Supplementary Figure 1: Comparison of mouse plasma cholesterol measurements from clinical chemistry analyzer and LC-MS/MS, respectively

CB57BL/6 WT and *Abcg*5/*8* DKO mice were fed PSRD for 3 weeks (n=15 in each group). Each individual plasma sample was divided into two aliquots for cholesterol measurement by (a) COBAS INTEGRA 400 plus clinical chemistry analyzer and by (b) LC-MS/MS, respectively. Data were analyzed using one-way ANOVA and presented using box-and-whisker plot, where the boxes encompass the first to the third quartiles, inside the box the horizontal line shows the median and the whiskers are the maximum and minimum observation.

Supplementary Figure 2

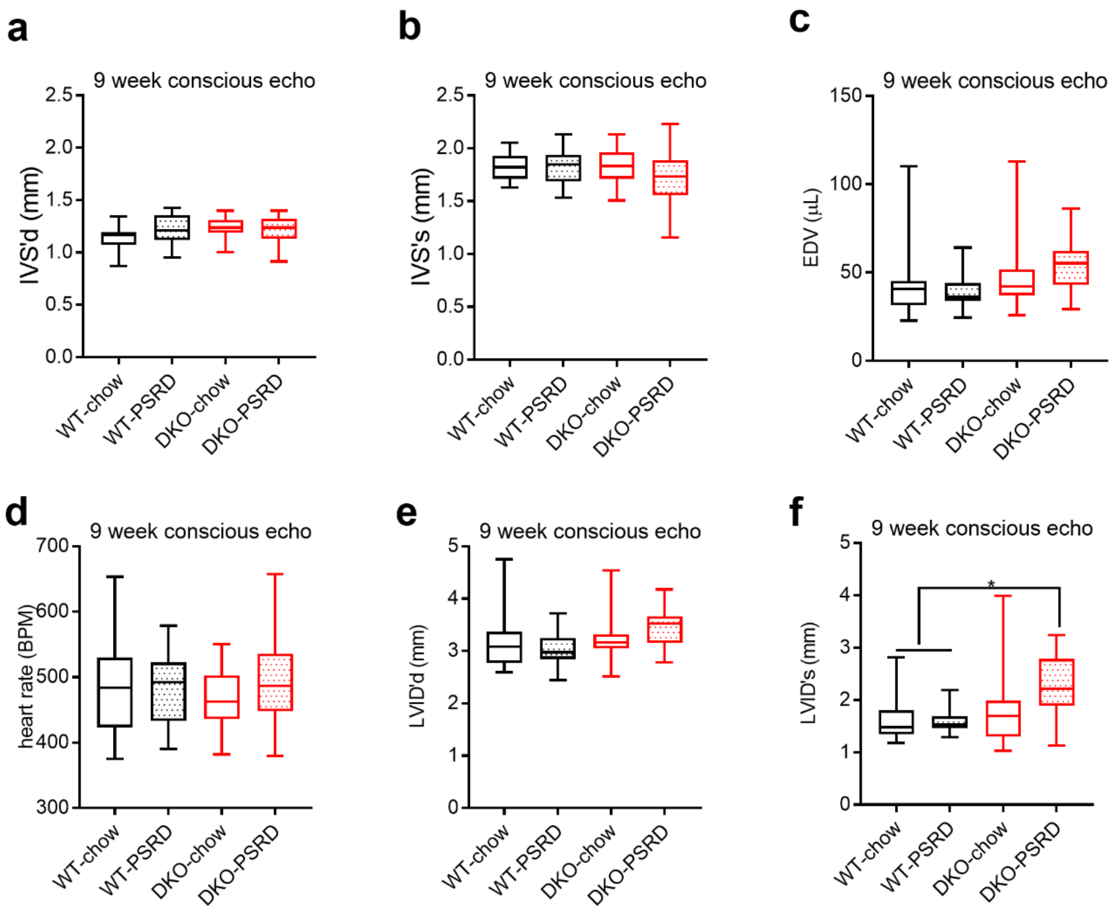

Supplementary Figure 2: Additional cardiac function measurements

Additional data collected from weeks 9 conscious echocardiogram. (a, b) interventricular septal (IVS'd and IVS's), (c) end diastolic volume (EDV), (d) heart rate, and (E, F) left ventricular internal diameter (LVID'd and LVID's) WT-chow (n=15), DKO-chow (n=13), WT-PSRD (n=16), DKO-PSRD (n=16) Data were analyzed using one-way ANOVA and presented using box-and-whisker plot, where the boxes encompass the first to the third quartiles, inside the box the horizontal line shows the median and the whiskers are the maximum and minimum observation. *p <0.05, **p <0.005, ***p <0.0001

Supplementary Figure 3

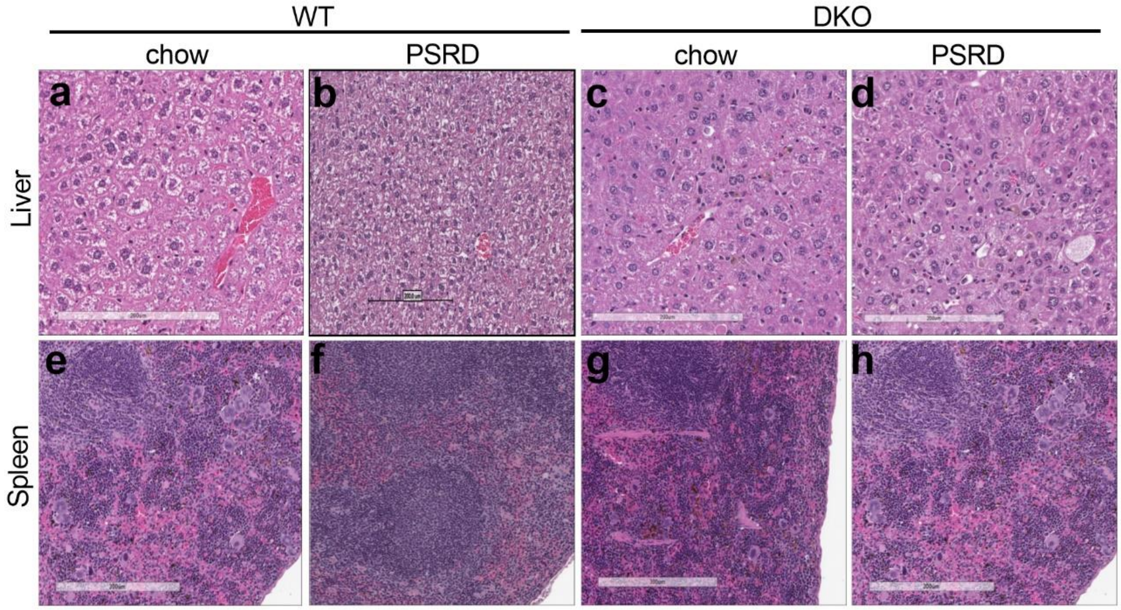

Supplementary Figure 3. No fibrosis is detected in liver and spleen
Images of H&E staining of the liver (a−d) and spleen (e−h) from a single representative section for each cohort are shown. Scale bar equals 200 µm (liver) and 300 µm (spleen)

Supplementary Figure 4

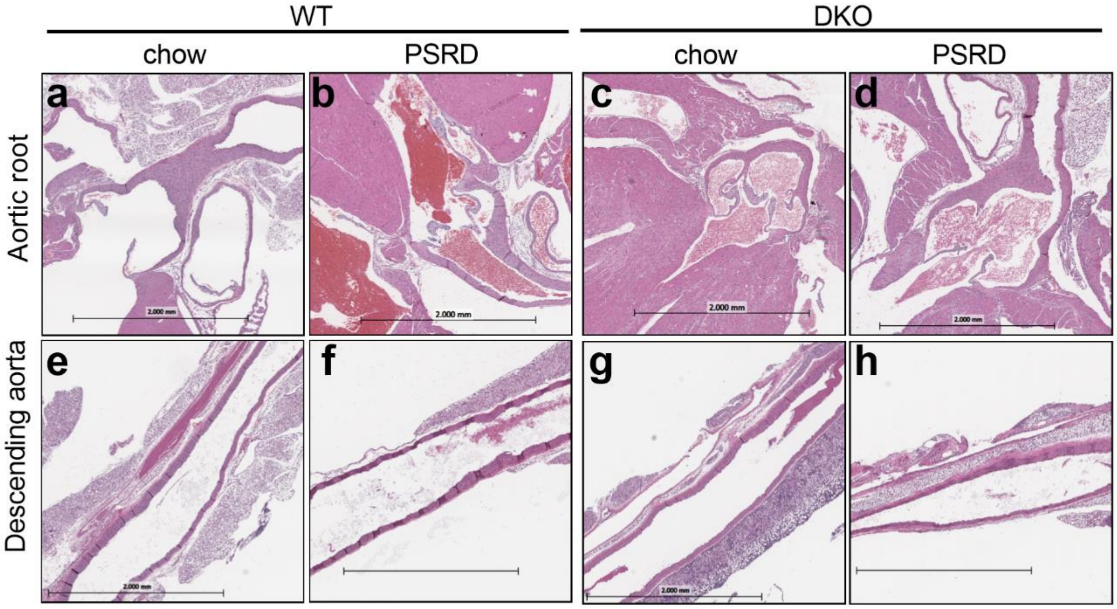

Supplementary Figure 4. Lack of atherosclerotic plaque formation or foam cells in the aorta of phytosterolemic mice

Images of H&E staining of the aortic roots (a–d) and thoracic aorta (e–h) from a single representative section for each cohort are shown. Scale bar equals 2 mm.

SUPPLEMENTARY TABLE 1

Table 1 Animal numbers at weeks 0 and 12 of study
No mouse died in WT-chow (n=15) or WT-PSRD (n=16) cohorts. Two deaths occurred in the DKO-chow (n=15) cohort and 10 deaths in the DKO-PSRD (n=25) cohort

Cohorts	Week 0 Enrolled	Week 12 Survived
WT-chow	15	15
WT-PSRD	16	16
DKO-chow	15	13
DKO-PSRD	25	15
